# Supplementary material for: A systematic review of post COVID-19 condition in children and adolescents: Gap in evidence from low-and -middle-income countries and the impact of SARS-COV-2 variants
Source: PLoS One. 2025 Mar 3;20(3):e0315815. doi: 10.1371/journal.pone.0315815 (PMC11875387; doi:10.1371/journal.pone.0315815)
Supplement: S1 File — (DOCX) [file pone.0315815.s001.docx]

Supplement 1. Search strategy

**Medline (from Ovid)**

30^th^ of October 2023

| 1. | COVID-19/ or SARS-CoV-2/ |
| --- | --- |
| 2. | (2019-novel or 2019nCoV or 2019-nCoV or COVID-19 or COVID19 or COVID-2019 or COVID2019 or CONVID-19 or CONVID19 or CORVID-19 or CORVID19 or CoV2 or CoV-2 or HCoV* or Ncov* or Ncorona* or Ncorono* or NcovChina* or NcovChinese* or NcovHubei* or NcovWuhan* or SARS2 or SARS-2 or SARScoronavirus2 or SARScoronavirus-2 or SARScoronovirus2 or SARScoronovirus-2 or SARSCov19 or SARSCov-19 or SARS-CoV-2 or SARSCoV-2 or SARSCoV2 or WN-CoV or WNCoV or wuhan-virus).tw,kf,ot. |
| 3. | ((new or novel or nouveau or "19" or "2019" or Wuhan or Hubei or Huanan or China or Chinese) adj3 (coronavirus* or corona virus* or betacoronavirus* or CoV or HCoV)).tw,kf,ot. |
| 4. | (coronavirus/ or betacoronavirus/ or coronavirus infections/) and (disease outbreaks/ or epidemics/ or pandemics/) |
| 5. | ((coronavirus* or corona-virus* or betacoronavirus*) adj3 (pandemic* or epidemic* or outbreak* or crisis)).tw,kf,ot. |
| 6. | 1 or 2 or 3 or 4 or 5 |
| 7. | (newborn* or new-born* or baby or babies or neonat* or neo-nat* or infan* or toddler* or pre-school* or preschool* or one-year-old* or one-years-old* or two-year-old* or two-years-old* or three-year-old* or three-years-old* or four-year-old* or four-years-old* or five-year-old* or five-years-old* or 1-year-old* or 1-years-old* or 2-year-old* or 2-years-old* or 3-year-old* or 3-years-old* or 4-year-old* or 4-years-old* or 5-year-old* or 5-years-old* or aged-one or aged-1 or aged-two or aged-2 or aged-three or aged-3 or aged-four or aged-4 or aged-five or aged-5 or less-than-5-years or less-than-five-years or younger-than-5-years or younger-than-five-years).af. |
| 8. | (pre-schooler* or preschooler* or kinder or kinders or kindergarten* or kinder-aged or boy or boys or girl or girls or child or children or childhood or pediatric* or paediatric* or school-age* or schoolage* or schoolchild* or schoolgirl* or schoolboy* or adolescen* or youth or youths or teen or teens or teenage*).af. |
| 9. | 7 or 8 |
| 10. | (longCOVID* or postCOVID* or postcoronavirus* or postSARS*).ti,ab,kf,ot. |
| 11. | (clinical-sequela* or long-term-symptom* or long-term-effect* or longterm-symptom* or longterm-effect* or persistent-symptom*).tw,kf. |
| 12. | 10 or 11 |
| 13. | 6 and 9 and 12 |

**351**

**Embase**

30^th^ of October 2023

| 1. | coronavirus disease 2019/ or SARS coronavirus/ or experimental coronavirus disease 2019/ or exp severe acute respiratory syndrome coronavirus 2/ |
| --- | --- |
| 2. | (2019-novel or 2019nCoV or 2019-nCoV or COVID-19 or COVID19 or COVID-2019 or COVID2019 or CONVID-19 or CONVID19 or CORVID-19 or CORVID19 or CoV2 or CoV-2 or HCoV* or Ncov* or Ncorona* or Ncorono* or NcovChina* or NcovChinese* or NcovHubei* or NcovWuhan* or SARS2 or SARS-2 or SARScoronavirus2 or SARScoronavirus-2 or SARScoronovirus2 or SARScoronovirus-2 or SARSCov19 or SARSCov-19 or SARS-CoV-2 or SARSCoV-2 or SARSCoV2 or WN-CoV or WNCoV or wuhan-virus).tw,kf,dq,ot. |
| 3. | ((new or novel or nouveau or "19" or "2019" or Wuhan or Hubei or Huanan or China or Chinese) adj3 (coronavirus* or corona virus* or betacoronavirus* or CoV or HCoV)).tw,kf,dq,ot. |
| 4. | (longCOVID* or postCOVID* or postcoronavirus* or postSARS*).ti,ab,kf,dq,ot. |
| 5. | severe-acute-respiratory-syndrome-coronavirus-2.hw. |
| 6. | coronavirus-disease-2019.hw. |
| 7. | (newborn* or new-born* or baby or babies or neonat* or neo-nat* or infan* or toddler* or pre-school* or preschool* or one-year-old* or one-years-old* or two-year-old* or two-years-old* or three-year-old* or three-years-old* or four-year-old* or four-years-old* or five-year-old* or five-years-old* or 1-year-old* or 1-years-old* or 2-year-old* or 2-years-old* or 3-year-old* or 3-years-old* or 4-year-old* or 4-years-old* or 5-year-old* or 5-years-old* or aged-one or aged-1 or aged-two or aged-2 or aged-three or aged-3 or aged-four or aged-4 or aged-five or aged-5 or less-than-5-years or less-than-five-years or younger-than-5-years or younger-than-five-years).tw. |
| 8. | (pre-schooler* or preschooler* or kinder or kinders or kindergarten* or kinder-aged or boy or boys or girl or girls or child or children or childhood or pediatric* or paediatric* or school-age* or schoolage* or schoolchild* or schoolgirl* or schoolboy* or adolescen* or youth or youths or teen or teens or teenage*).af. |
| 9. | (clinical-sequela* or long-term-symptom* or long-term-effect* or longterm-symptom* or longterm-effect*).ti,ab,kf,dq,ot. |
| 10. | 1 or 2 or 3 or 5 |
| 11. | 4 or 9 |
| 12. | 7 or 8 |
| 13. | 10 and 11 and 12 |

**317**

**PubMed**

30^th^ of October 2023

# 1 All fields

“2019-novel” OR “2019nCoV” OR “2019-nCoV” OR “COVID-19” OR “COVID19” OR “COVID-2019” OR “COVID2019” OR “CONVID-19” OR “CONVID19” OR “CORVID-19” OR “CORVID19” OR “CoV2” OR “CoV-2” OR “HCoV*” OR “Ncov*” OR “Ncorona*” OR “Ncorono*” OR “NcovChina*” OR “NcovChinese*” OR “NcovHubei*” OR “NcovWuhan*” OR “SARS2” OR “SARS-2” OR “SARScoronavirus2” OR “SARScoronavirus-2” OR “SARScoronovirus2” OR “SARScoronovirus-2” OR “SARSCov19” OR “SARSCov-19” OR “SARS-CoV-2” OR “SARSCoV-2” OR “SARSCoV2” OR “WN-CoV” OR “WNCoV” OR “wuhan-virus”

#2 All fields

(“pneumonia*” OR “outbreak*” OR “respiratory-illness*” OR “respiratory-disease*” OR “respiratory-symptom*” OR “seafood-market*” OR “food-market*” OR “wildlife”) AND (“Wuhan” OR “China” OR “Chinese” OR “Hubei” OR “Huanan”)

#3 All fields

(“new” OR “novel” OR “nouveau” OR “19” OR “2019” OR “Wuhan” OR “Hubei” OR “Huanan” OR “China” OR “Chinese”) AND (“coronavirus*” OR “corona virus*” OR “betacoronavirus*” OR “CoV” OR “HCoV”)

#4 All fields

“longCOVID*” OR “postCOVID*” OR “postcoronavirus*” OR “postSARS*”

#5 All fields

(“coronavirus*” OR “corona-virus*” OR “betacoronavirus*”) AND (“pandemic*” OR “epidemic*” OR “outbreak*” OR “crisis”)

Filter:

Child: birth-18 years, Newborn: birth-1 month, Infant: birth-23 months, Infant: 1-23 months, Child: 6-12 years, Adolescent: 13-18 years, Preschool Child: 2-5 years*.*

**283**

**Identification of studies via other methods**

13^th^ of February 2024

27
