# Supplementary material for: A systematic review of post COVID-19 condition in children and adolescents: Gap in evidence from low-and -middle-income countries and the impact of SARS-COV-2 variants
Source: PLoS One. 2025 Mar 3;20(3):e0315815. doi: 10.1371/journal.pone.0315815 (PMC11875387; doi:10.1371/journal.pone.0315815)
Supplement: S2 Table — (DOCX) [file pone.0315815.s002.docx]

**Supplementary 2. Quality of the study**

| **STROBE checklist for observational study** | **Adler et al** | **Ahn et al** | **Asadi-Pooya** | **Ashkenazi-Hoffnung** | **Baptista de lima et al** | **Bergia** | **Bloise** | **Blomberg et al** | **Borch** | **Bossley et al** | **Brackel** | **Buonsenso** | **Buonsenso (2)** | **Buonsenso c et al** | **Erol et al** | **Fink** | **Funk** | **Gonzales et al** | **Haddad** | **Kikkenborg Berg** | **Knoke** | **Kompaniyets** | **Kuczborska** | **Matteudi et al** | **Miller et al** | **Molteni** | **Osmanov** | **Pazukhina** | **Pereira et al** | **Radtke** | **Roge** | **Sakurada et al** | **Say** | **Seery et al** | **Smane (1)** | **Smane (2)** | **Stephenson** | **Sterky** | **Trapani et al** | **Zavala** |
| --- | --- | --- | --- | --- | --- | --- | --- | --- | --- | --- | --- | --- | --- | --- | --- | --- | --- | --- | --- | --- | --- | --- | --- | --- | --- | --- | --- | --- | --- | --- | --- | --- | --- | --- | --- | --- | --- | --- | --- | --- |
| **Title and abstract** |  |  |  |  |  |  |  |  |  |  |  |  |  |  |  |  |  |  |  |  |  |  |  |  |  |  |  |  |  |  |  |  |  |  |  |  |  |  |  |  |
| **Introduction** | | | | | | | | | | | | | | | | | | | | | | | | | | | | | | | | | | | | | | | | |
| Background |  |  |  |  |  |  |  |  |  |  |  |  |  |  |  |  |  |  |  |  |  |  |  |  |  |  |  |  |  |  |  |  |  |  |  |  |  |  |  |  |
| Objectives |  |  |  |  |  |  |  |  |  |  |  |  |  |  |  |  |  |  |  |  |  |  |  |  |  |  |  |  |  |  |  |  |  |  |  |  |  |  |  |  |
| **Methods** | | | | | | | | | | | | | | | | | | | | | | | | | | | | | | | | | | | | | | | | |
| Study design |  |  |  |  |  |  |  |  |  |  |  |  |  |  |  |  |  |  |  |  |  |  |  |  |  |  |  |  |  |  |  |  |  |  |  |  |  |  |  |  |
| Setting |  |  |  |  |  |  |  |  |  |  |  |  |  |  |  |  |  |  |  |  |  |  |  |  |  |  |  |  |  |  |  |  |  |  |  |  |  |  |  |  |
| Participants |  |  |  |  |  |  |  |  |  |  |  |  |  |  |  |  |  |  |  |  |  |  |  |  |  |  |  |  |  |  |  |  |  |  |  |  |  |  |  |  |
| Variables |  |  |  |  |  |  |  |  |  |  |  |  |  |  |  |  |  |  |  |  |  |  |  |  |  |  |  |  |  |  |  |  |  |  |  |  |  |  |  |  |
| Data sources/  Measurement |  |  |  |  |  |  |  |  |  |  |  |  |  |  |  |  |  |  |  |  |  |  |  |  |  |  |  |  |  |  |  |  |  |  |  |  |  |  |  |  |
| Bias |  |  |  |  |  |  |  |  |  |  |  |  |  |  |  |  |  |  |  |  |  |  |  |  |  |  |  |  |  |  |  |  |  |  |  |  |  |  |  |  |
| Study size |  |  |  |  |  |  |  |  |  |  |  |  |  |  |  |  |  |  |  |  |  |  |  |  |  |  |  |  |  |  |  |  |  |  |  |  |  |  |  |  |
| Quantitative variables |  |  |  |  |  |  |  |  |  |  |  |  |  |  |  |  |  |  |  |  |  |  |  |  |  |  |  |  |  |  |  |  |  |  |  |  |  |  |  |  |
| Statistical methods |  |  |  |  |  |  |  |  |  |  |  |  |  |  |  |  |  |  |  |  |  |  |  |  |  |  |  |  |  |  |  |  |  |  |  |  |  |  |  |  |
| **Results** | | | | | | | | | | | | | | | | | | | | | | | | | | | | | | | | | | | | | | | | |
| Participants |  |  |  |  |  |  |  |  |  |  |  |  |  |  |  |  |  |  |  |  |  |  |  |  |  |  |  |  |  |  |  |  |  |  |  |  |  |  |  |  |
| Descriptive data |  |  |  |  |  |  |  |  |  |  |  |  |  |  |  |  |  |  |  |  |  |  |  |  |  |  |  |  |  |  |  |  |  |  |  |  |  |  |  |  |
| Outcome data |  |  |  |  |  |  |  |  |  |  |  |  |  |  |  |  |  |  |  |  |  |  |  |  |  |  |  |  |  |  |  |  |  |  |  |  |  |  |  |  |
| Main results |  |  |  |  |  |  |  |  |  |  |  |  |  |  |  |  |  |  |  |  |  |  |  |  |  |  |  |  |  |  |  |  |  |  |  |  |  |  |  |  |
| Other analysis |  |  |  |  |  |  |  |  |  |  |  |  |  |  |  |  |  |  |  |  |  |  |  |  |  |  |  |  |  |  |  |  |  |  |  |  |  |  |  |  |
| **Discussion** | | | | | | | | | | | | | | | | | | | | | | | | | | | | | | | | | | | | | | | | |
| Key results |  |  |  |  |  |  |  |  |  |  |  |  |  |  |  |  |  |  |  |  |  |  |  |  |  |  |  |  |  |  |  |  |  |  |  |  |  |  |  |  |
| Limitations |  |  |  |  |  |  |  |  |  |  |  |  |  |  |  |  |  |  |  |  |  |  |  |  |  |  |  |  |  |  |  |  |  |  |  |  |  |  |  |  |
| Interpretation |  |  |  |  |  |  |  |  |  |  |  |  |  |  |  |  |  |  |  |  |  |  |  |  |  |  |  |  |  |  |  |  |  |  |  |  |  |  |  |  |
| Generalisability |  |  |  |  |  |  |  |  |  |  |  |  |  |  |  |  |  |  |  |  |  |  |  |  |  |  |  |  |  |  |  |  |  |  |  |  |  |  |  |  |
| **Other information** | | | | | | | | | | | | | | | | | | | | | | | | | | | | | | | | | | | | | | | | |
| Funding |  |  |  |  |  |  |  |  |  |  |  |  |  |  |  |  |  |  |  |  |  |  |  |  |  |  |  |  |  |  |  |  |  |  |  |  |  |  |  |  |
|  | H | L | M | L | M | M | M | M | M | L | L | M | M | M | M | M | H | H | H | H | M | M | L | M | H | H | M | H | H | L | H | M | L | H | L | L | H | L | H | M |

H: high; M: medium; L: low

| Adherence to STROBE recommendation | Yes |  |
| --- | --- | --- |
|  | No |  |
|  | Partial |  |

| **STROBE checklist for observational study** | **Adler et al** | **Ahn et al** | **Asadi-Pooya** | **Ashkenazi-Hoffnung** | **Baptista de lima et al** | **Bergia** | **Bloise** | **Blomberg et al** | **Borch** | **Bossley et al** | **Brackel** | **Buonsenso** | **Buonsenso (2)** | **Buonsenso c et al** | **Erol et al** | **Fink** | **Funk** | **Gonzales et al** | **Haddad** | **Kikkenborg Berg** | **Knoke** | **Kompaniyets** | **Kuczborska** | **Matteudi et al** | **Miller et al** | **Molteni** | **Osmanov** | **Pazukhina** | **Pereira et al** | **Radtke** | **Roge** | **Sakurada et al** | **Say** | **Seery et al** | **Smane (1)** | **Smane (2)** | **Stephenson** | **Sterky** | **Trapani et al** | **Zavala** |
| --- | --- | --- | --- | --- | --- | --- | --- | --- | --- | --- | --- | --- | --- | --- | --- | --- | --- | --- | --- | --- | --- | --- | --- | --- | --- | --- | --- | --- | --- | --- | --- | --- | --- | --- | --- | --- | --- | --- | --- | --- |
| **Scoring** | **43** | **27** | **33** | **26** | **31** | **36** | **35** | **34** | **34** | **29** | **25** | **38** | **35** | **36** | **37** | **32** | **44** | **44** | **42** | **44** | **38** | **38** | **17** | **37** | **40** | **42** | **38** | **42** | **42** | **26** | **42** | **33** | **21** | **41** | **27** | **27** | **42** | **25** | **40** | **38** |

Categories of the quality of studies:

- Low (score ≤ 29)
- Moderate (score 30–39)
- High quality (score ≥40).
