# Supplementary material for: A systematic review of post COVID-19 condition in children and adolescents: Gap in evidence from low-and -middle-income countries and the impact of SARS-COV-2 variants
Source: PLoS One. 2025 Mar 3;20(3):e0315815. doi: 10.1371/journal.pone.0315815 (PMC11875387; doi:10.1371/journal.pone.0315815)
Supplement: S3 Table — (DOCX) [file pone.0315815.s003.docx]

**Supplementary 3. Additional Study Details**

|  | Study | Setting | GNI per country (by October 2023) | Economy Group | Symptoms time course / definition of post COVID-19 condition | Aligned with WHO definition 2023 |
| --- | --- | --- | --- | --- | --- | --- |
| 1. | Adler et al | Israel | 54,650 | HIC | >4 weeks after diagnosis | No |
| 2. | Ahn et al | Korea | 35,990 | HIC | >12 weeks after diagnosis | Yes |
| 3. | Asadi-Pooya et al | Iran | 3,900 | LMIC | >3 months from diagnosis | Yes |
| 4. | Ashkenazi-Hoffnung, et al | Israel | 54,650 | HIC | >4 weeks from diagnosis | No |
| 5. | Baptista de lima et al | Portugal | 25,800 | HIC | >4–24 weeks after diagnosis | Yes |
| 6. | Bergia et al. | Spain | 31,680 | HIC | >12 weeks from diagnosis | Yes |
| 7. | Blomberg et al | Norway | 95,510 | HIC | 6 months after diagnosis | Yes |
| 8. | Bloise, et al | Italy | 37,700 | HIC | N/A | No |
| 9. | Borch, et al | Denmark | 73,200 | HIC | >4 weeks from diagnosis | No |
| 10. | Bossley et al | UK | 48,890 | HIC | >4 weeks after diagnosis | No |
| 11. | Brackel, et al | Netherlands | 57,430 | HIC | >12 weeks from diagnosis | Yes |
| 12. | Buonsenso, et al (a) | Italy | 37,700 | HIC | > 4 weeks from diagnosis | No |
| 13. | Buonsenso, et al (b) | UK | 48,890 | HIC | >4 weeks from diagnosis | No |
| 14. | Buonsenso c et al | Italy | 37,700 | HIC | >60–120 days after diagnosis | Yes |
| 15. | Erol et al | Turkey | 10,590 | UMIC | >1month – 1 year after diagnosis | Yes |
| 16. | Fink, et al | Brazil | 8,140 | UMIC | >12 weeks from diagnosis | Yes |
| 17. | Funk, et al | Argentina  Canada  Costa Rica  Italy  Paraguay  Singapore  Spain  USA | 11,620  52,960  12,670  37,700  5,920  67,200  31,680  76,370 | UMIC and HIC | 90–120 days after ED visit | Yes |
| 18. | Gonzales et al | Spain | 31,680 | HIC | >12 weeks after diagnosis | Yes |
| 19. | Haddad, et al | Germany | 53,590 | HIC | 11–12 months after diagnosis | Yes |
| 20. | Kikkenborg Berg, et al | Denmark | 73,200 | HIC | ≥2 months after diagnosis | Yes |
| 21. | Knoke, et al | Germany | 53,590 | HIC | ≥4 weeks after acute infection | No |
| 22. | Kompaniyets, et al | USA | 76,370 | HIC | >60–365 days after diagnosis | Yes |
| 23. | Kuczborska et al | Poland | 18,350 | HIC | >12 weeks after diagnosis | Yes |
| 24. | Matteudi et al | France | 45,860 | HIC | 10–13 months after diagnosis | Yes |
| 25. | Molteni, et al | UK | 48,890 | HIC | 56 days after diagnosis | Yes |
| 26. | Miller et al | UK and Wales | 48,890 | HIC | ≥4 weeks after diagnosis | No |
| 27. | Osmanov, et al | Russia | 12,830 | UMIC | >5 months after hospitalization | Yes |
| 28. | Pazukhina, et al | Russia | 12,830 | UMIC | >6 months after hospital discharge | Yes |
| 29. | Pereira et al | UK | 48,890 | HIC | >6 months after diagnosis | Yes |
| 30. | Radtke, et al | Switzerland | 89,450 | HIC | >6 months after diagnosis | Yes |
| 31. | Roge, et al | Latvia | 21,500 | HIC | >1–6 months after diagnosis | Yes |
| 32. | Sakurada et al | Japan | 42,440 | HIC | >4 weeks after diagnosis | No |
| 33. | Say, et al | Australia | 60,430 | HIC | >3–6 months after diagnosis | Yes |
| 34. | Seery et al | Argentina | 11,620 | UMIC | >3 months after diagnosis | Yes |
| 35. | Smane et al (1) | Latvia | 21,500 | HIC | N/A | No |
| 36. | Smane, et al (2) | Latvia | 21,500 | HIC | 1–3 months after onset | No |
| 37. | Stephenson, et al | UK | 48,890 | HIC | 3 months after diagnosis | Yes |
| 38. | Sterky, et al | Sweden | 62,990 | HIC | 4 months after hospital admission | Yes |
| 39. | Trapani et al. | Italy | 37,700 | HIC | 2–9 months after recovery of acute infection | Yes |
| 40. | Zavala et al. | UK | 48,890 | HIC | >4 weeks after diagnosis | No |
