# Supplementary material for: A systematic review of post COVID-19 condition in children and adolescents: Gap in evidence from low-and -middle-income countries and the impact of SARS-COV-2 variants
Source: PLoS One. 2025 Mar 3;20(3):e0315815. doi: 10.1371/journal.pone.0315815 (PMC11875387; doi:10.1371/journal.pone.0315815)
Supplement: S6 Table — (DOCX) [file pone.0315815.s006.docx]

Supplementary 6. Details of Excluded Studies

From electronic search

| No. | Author | Rason of exclusion | Link |
| --- | --- | --- | --- |
| 1. | Fainardi, et al. | Systematic review | https://pubmed.ncbi.nlm.nih.gov/35207572/ |
| 2. | Behnood, et al. | Systematic review | https://pubmed.ncbi.nlm.nih.gov/34813820/ |
| 3. | Franco, et al. | Systematic review | https://pubmed.ncbi.nlm.nih.gov/36361269/ |
| 4. | Lopez-Leon, et al. | Systematic review | https://www.nature.com/articles/s41598-022-13495-5 |
| 5. | Pellegrino, et al | Systematic review | https://pubmed.ncbi.nlm.nih.gov/36107254/ |
| 6. | Zheng, et al | Systematic review | https://pubmed.ncbi.nlm.nih.gov/36931142/ |
| 7. | Zimmermann, et al. | Systematic review | https://pubmed.ncbi.nlm.nih.gov/34870392/ |
| 8. | Jiang, et al. | Systematic review | https://publications.aap.org/pediatrics/article/152/2/e2022060351/192816/A-Systematic-Review-of-Persistent-Clinical?autologincheck=redirected |
| 9. | Sansone, et al. | Review | https://www.mdpi.com/2075-4418/13/12/1990 |
| 10. | Gupta, et al. | Review | https://pubmed.ncbi.nlm.nih.gov/35486940/ |
| 11. | Yong, et al. | Review | https://pubmed.ncbi.nlm.nih.gov/34024217/ |
| 12. | Thallapureddy, et al. | Review | https://pubmed.ncbi.nlm.nih.gov/35127274/ |
| 13. | Howard-Jones, et al. | Review | https://pmc.ncbi.nlm.nih.gov/articles/PMC8662268/ |
| 14. | Izquierdo-Pujol | Review | https://pmc.ncbi.nlm.nih.gov/articles/PMC9130634/ |
| 15. | Yonts | Review | https://pubmed.ncbi.nlm.nih.gov/36343180/ |
| 16. | Filippatos, et al. | Review | https://pmc.ncbi.nlm.nih.gov/articles/PMC9468832/ |
| 17. | Piazza, et al. | Review | https://pmc.ncbi.nlm.nih.gov/articles/PMC9024951/ |
| 18. | Ehrler, et al. | Specific symptoms (well-being and family functioning) | https://pubmed.ncbi.nlm.nih.gov/33486835/ |
| 19. | Bussieres, et al. | Specific symptoms (mental health) | https://pubmed.ncbi.nlm.nih.gov/34925080/ |
| 20. | Rathgeb, et al. | Specific symptoms (psychological) | https://www.mdpi.com/1660-4601/19/5/2698 |
| 21. | Dobkin, et al | Specific symptoms (respiratory findings only) | https://www.atsjournals.org/doi/10.1164/ajrccm-conference.2021.203.1_MeetingAbstracts.A3404 |
| 22. | Logan, et al. | Specific symptoms (mental health, sleep, and physical activity) | https://www.neurology.org/doi/10.1212/WNL.96.15_supplement.2876 |
| 23. | Denina, et al. | Specific only laboratory and lung ultrasound follow up | https://pubmed.ncbi.nlm.nih.gov/33003103/ |
| 24. | Buonsenso, et al. | Specific symptoms (anosmia) | https://pubmed.ncbi.nlm.nih.gov/36010141/ |
| 25. | Tabacof, et al. | Specific symptoms (Physical Function, Cognitive Function, Health-Related Quality of Life, and Participation) | https://pubmed.ncbi.nlm.nih.gov/34686631/ |
| 26. | Vasichkina, et al. | Case Report | https://pmc.ncbi.nlm.nih.gov/articles/PMC10056761/ |
| 27. | Warschburger, et al. | Protocol | https://pubmed.ncbi.nlm.nih.gov/36949465/ |
| 28. | Parisi, et al. | Not relevant (pediatrician-reported persistent symptoms) | https://pmc.ncbi.nlm.nih.gov/articles/PMC8467017/pdf/children-08-00769.pdf |

From additional search

| No. | Author | Reason of exclusion | Link |
| --- | --- | --- | --- |
| 1. | Nogueira López, et al. | Sample size <20 | https://pmc.ncbi.nlm.nih.gov/articles/PMC8251185/pdf/APA-110-2282.pdf |
| 2. | Demiburga, et al. | Not retrieved, in Turkish | https://pubmed.ncbi.nlm.nih.gov/36636846/ |
| 3. | Ertesvåg N et al. | Population do not match (include adults) | https://pmc.ncbi.nlm.nih.gov/articles/PMC10166589/ |
| 4. | Khoury J et al. | Population do not match (self reported diagnosis only) | https://www.ncbi.nlm.nih.gov/pmc/articles/PMC10356177/ |
| 5. | Maddux, et al. | Population do not match (include adults) | https://pubmed.ncbi.nlm.nih.gov/35765138/ |
| 6. | Messiah, et al. | Population do not match (include adults) | https://pubmed.ncbi.nlm.nih.gov/35939608/ |
| 7. | Zayet, et al. | Population do not match (adults) | https://www.mdpi.com/2076-2607/9/8/1719 |
| 8. | Fernández-de-Las-Peñas, et al. | Population do not match (adults) | https://pubmed.ncbi.nlm.nih.gov/36560633/ |
| 9. | Saigal, et al. | Population do not match (adults) | https://pubmed.ncbi.nlm.nih.gov/37536948/ |
