# Supplementary material for: A systematic review of post COVID-19 condition in children and adolescents: Gap in evidence from low-and -middle-income countries and the impact of SARS-COV-2 variants
Source: PLoS One. 2025 Mar 3;20(3):e0315815. doi: 10.1371/journal.pone.0315815 (PMC11875387; doi:10.1371/journal.pone.0315815)
Supplement: S7 Table — (DOCX) [file pone.0315815.s007.docx]

Supplementary 7. Data Extraction Details

| No. | Data | Extractor | Extraction date | Verificator that study was eligible |
| --- | --- | --- | --- | --- |
| 1. | Study details (author, time of data collection, study location, study design) | NDP | 31^st^ of October –8^th^ of November 2023 | SMG and JEB |
| 2. | Participant details (number of subjects, sex, age of overall participants, age of participants in post COVID-19 condition group and control if any, acute SARS-CoV-2 confirmation method, the severity of acute COVID-19) | NDP | 31^st^ of October –8^th^ of November 2023 | SMG and JEB |
| 3. | Outcome details (post COVID-19 condition definition, duration of follow up, post COVID-19 symptom prevalence, and the three most frequently reported persistent symptoms with prevalence of each | NDP | 31^st^ of October –8^th^ of November 2023 | SMG and JEB |
