# Supplementary material for: A systematic review of post COVID-19 condition in children and adolescents: Gap in evidence from low-and -middle-income countries and the impact of SARS-COV-2 variants
Source: PLoS One. 2025 Mar 3;20(3):e0315815. doi: 10.1371/journal.pone.0315815 (PMC11875387; doi:10.1371/journal.pone.0315815)
Supplement: Attachment — Submitted filename: PONE-D-24-06176-IR.docx [file pone.0315815.s008.docx]

**A review of the manuscript entitled “A systematic review of post COVID-19 condition in children and adolescents: Gap in evidence from low- and middle-income countries and the impact of SARS-COV-2 variants”**

1. Overall, this manuscript has given a clear and relevant explanation regarding the impact of SARS-CoV-2 variants in children and adolescence from HICs, UMICs, and LMICs. The manuscript is scientifically sound and the experimental design is reproducible. The data is interpreted appropriately throughout the manuscript. However, I have highlighted a room for improvement, which can be found below. Please address them accordingly. Thank you!
2. (page 10, line 38): What does it mean with “1.8 to 70%”? Please unify the usage of percentage symbol to each mentioned numerical value.
3. (page 10, lines 40-42): The repetition of “when Alpha predominated” from this sentence should be removed.
4. (page 11, lines 61-62): This sentence “A systematic review of post COVID-19 condition indicates . .” will be more significant if it’s also supported by another relevant study. Please check <https://doi.org/10.52225/narra.v1i3.48>
5. (page 12, line 101): What is the meaning of “tw, kf, dq, and hw”? Please explain.
6. Please make sure that the percentage symbol is used with similar format throughout the manuscript. I noticed that the authors inconsistently use this symbol.
7. Authors are suggested to proofread the manuscript after addressing all comments to avoid any typo, grammatical, and lingual mistakes and errors.
8. (page 37, figure 4): Please make sure to apply the correct writing of SARS-CoV-2.
9. (page 24, lines 358-360): This sentence “There are currently no published data . . .” is such a bold claim because I found a literature that reported a case of co-infection between SARS-CoV-2 and *Orientia tsutsugamushi* in a young teen in Nepal. Please check Bastola A, Sah R, Rajbhandari SK, et al. SARS-CoV-2 and Orientia tsutsugamushico-infection in a young teen, Nepal: Significant burden in limited-resource countries in Asia?
