# Supplementary material for: A systematic review of post COVID-19 condition in children and adolescents: Gap in evidence from low-and -middle-income countries and the impact of SARS-COV-2 variants
Source: PLoS One. 2025 Mar 3;20(3):e0315815. doi: 10.1371/journal.pone.0315815 (PMC11875387; doi:10.1371/journal.pone.0315815)
Supplement: Attachment — Submitted filename: Response to reviewers 271024.docx [file pone.0315815.s009.docx]

**Rebuttal letter**

Dear Editors,

We thank the reviewers for their generous comments on the manuscript and have edited it to address the concerns. The correction details are presented below.

We believe that the manuscript is now suitable for publication in PLOS One.

Sincerely,

Nina Dwi Putri

Reviewer 1.

1. Recent studies have shown the possible persistence of long COVID for 2 years in the general population (DOI: 10.1016/j.jinf.2023.12.004). However, the meta-analysis data reported in this study is only limited to the adult population. It would be important to emphasize the need for a more in-depth analysis looking at the prevalence of long COVID symptoms in the pediatric age group and how these symptoms compare with the adult population.

Response: Thanks for this important point. Specific reference to the adult data has been added in the Introduction, Lines 72-74. Emphasis of the need for more in-depth analysis in children with comparison to adult data is added in Discussion, with reference added to findings in studies that did include and report on adults in same population (refs 39 and 43). Lines 388-391.

2. Kindly include a supplementary section detailing the search strategy and the outcome of the meshed search terms for every journal database used.

Response: Thank you. This has now been added to Supplementary 1

3. Please include a PICO statement as well as the PRISMA checklist.

Response: A PICO statement has not been included as the data reviewed are observational. The Population line 120-124 and Outcomes line 127-130 are clearly defined and included. However, there is no Intervention being studied and so no Comparison can be made such as between age groups because of study heterogeneity. The characteristics of control groups (by age and sex) whenever reported are listed in Table 1 for the studies that compared persistence of symptoms to COVID-confirmed cases.

PRISMA Checklists have been added, which are:

- PRISMA Checklist for abstract (Supplementary 4)
- PRISMA Checklist for manuscript (Supplementary 5)

4. Kindly include the rationale for why a meta-analysis was not performed in this study. You can include this statement in the methods section.

Response: It was not possible to conduct a meta-analysis for reasons outlined in Methods line 226-229 which reads “We did not conduct a meta-analysis due to the wide heterogeneity in study design (such as varying definitions of post COVID-19 condition, timing and method of follow-up assessments) and in study participants (including variations in reporting by age groups and the severity of acute COVID).”

5. Did the studies included in the systematic review undergo quality assessment, such as using the Newcastle-Ottawa scale, QUIPS, etc.? If not, please include a quality check for all the studies to assess the risk of bias.

Response: There is a specific section in manuscript Line 187-188 under the sub-heading of “Assessment of Risk of Bias”, with detailed table included as Supplementary 2.

6. This systematic review lacks in-depth analysis. It is important to report the pooled demographics of the children and adolescents included in this review.

Response: Within the acknowledged limitations of the variation in reporting of data by the included studies, we have strived to report pooled demographics based on the data we extracted for participant details including by age, sex, definition, symptoms, diagnostic method and severity. Results paragraph: lines 245-251

7. Were there control groups included in the study? For example, children or adolescents who are either healthy or those who succumbed to an acute infection but did not develop long COVID?

Response: Thank you. We included all studies (with or without control) and the data regarding the control population when available are included in Table 1 such as age, sex and proportion with persistent symptoms when available. The statement can be found in Line 175-176.

8. What were the limitations of the present study? What are the gaps that need to be addressed in long COVID research involving these age groups?

Response: The limitations of available data are referred to repeatedly in the manuscript – the main ones being study heterogeneity not allowing meta-analysis and lack of clearly defined controls in many studies. Limitation of the included studies are presented separately in Line 374-3677 Line 378-380, Line 396-398, Line 425-428, line 434-437 while limitation of this review is shown in Line 425-4228 Line 444-448, Line 452-456, Line 456-458.

9. There are many theories contributing to the development of long COVID. I think it is important to highlight these studies, which include the possible persistence of SARS-CoV-2 RNA (DOI: 10.1515/cclm-2024-0036), excessive inflammatory response, and even the generation and persistence of autoantibodies (DOI: 10.3389/fimmu.2024.1428645).

Response: Thank you. These have been added to the manuscript. Line 392-398, Line 401-404, Line 416-424

10. It is also important to highlight the several factors that can influence the severity and risk of developing long COVID, including vaccination prior to an acute infection (DOI: 10.1016/j.eclinm.2022.101624), administration of antivirals during the acute phase of SARS-CoV-2 infection (DOI: 10.1007/s15010-023-02154-0), and demographics like sex and certain comorbidities (DOI: 10.3390/jcm11247314), as well as the infecting SARS-CoV-2 variant (DOI: 10.3390/v14122629).

Response: Thanks for these suggestions. The following have been added -

- Vaccination and SARS-CoV-2 variant has been added to Line 440-443
- Antiviral treatment has been added to Line 405-407
- Sex has been added to Line 444-452

We did not document comorbidities of included studies’ participants therefore did not discuss the influence of comorbidities to post COVID-19 condition

Reviewer 2.

11. Overall, this manuscript has given a clear and relevant explanation regarding the impact of SARS-CoV-2 variants in children and adolescence from HICs, UMICs, and LMICs. The manuscript is scientifically sound and the experimental design is reproducible. The data is interpreted appropriately throughout the manuscript. However, I have highlighted a room for improvement, which can be found below. Please address them accordingly. Thank you!

Response: thanks for this positive review.

12. (page 10, line 38): What does it mean with “1.8 to 70%”? Please unify the usage of percentage symbol to each mentioned numerical value.

Response: The format has been changed to 1.8% to 70%. All other percentages have also been changed to this format.

13. (page 10, lines 40-42): The repetition of “when Alpha predominated” from this sentence should be removed.

Response: This has been done. The line has been changed to: “The range of prevalence of reported post COVID-19 condition was 4.1%–66.7% in the studies in which the variant of concern was not identifiable, [45, 56] 6.7%–70% in the Alpha variant predominated studies, [62, 71] 23%–61.9% in the Delta predominated studies, [45, 49] 17–34.6% in the Omicron predominated studies, [33, 54] and 3.7%–34% in the Other-variant predominated studies [61,65] Fatigue was the commonest symptom reported in all variant subgroups (9/11 [81.8%] in Alpha, 4/4 [100%] in Delta, 2/4 [50%] in Omicron, 6/6 [100%] in Other-variant predominated studies, and 7/14 [50%] in unidentifiable-variant-predominated studies).”

14. (page 11, lines 61-62): This sentence “A systematic review of post COVID-19 condition indicates . .” will be more significant if it’s also supported by another relevant study. Please check <https://doi.org/10.52225/narra.v1i3.48>

Response: The study data are now referred to as: “A meta-analysis of 22 studies found that prolonged fatigue, joint pain, anosmia, headache and myalgia were recorded in 21.2%, 15.4%, 9.7%, 8.9% and 5.6% of adult COVID-19 survivors, respectively”.

15. (page 12, line 101): What is the meaning of “tw, kf, dq, and hw”? Please explain.

Response: now spelt out in text.

16. Please make sure that the percentage symbol is used with similar format throughout the manuscript. I noticed that the authors inconsistently use this symbol.

Response: All percentage format has been changed to “xx% to xx%

17. Authors are suggested to proofread the manuscript after addressing all comments to avoid any typo, grammatical, and lingual mistakes and errors.

Response: thank you for your feedback

18. (page 37, figure 4): Please make sure to apply the correct writing of SARS-CoV-2.

Response: it has been changed to: “**Fig 4.** **Distribution of Predominating SARS-CoV-2 Variants between Included Studies”**

19. (page 24, lines 358-360): This sentence “There are currently no published data . . .” is such a bold claim because I found a literature that reported a case of co-infection between SARS-CoV-2 and *Orientia tsutsugamushi* in a young teen in Nepal. Please check Bastola A, Sah R, Rajbhandari SK, et al. SARS-CoV-2 and Orientia tsutsugamushico-infection in a young teen, Nepal: Significant burden in limited-resource countries in Asia?

Response: thank you – now omitted

20. Please ensure that your manuscript meets PLOS ONE's style requirements, including those for file naming. The PLOS ONE style templates can be found at

Answer: we have followed the styling guideline

<https://journals.plos.org/plosone/s/file?id=wjVg/PLOSOne_formatting_sample_main_body.pdf> and <https://journals.plos.org/plosone/s/file?id=ba62/PLOSOne_formatting_sample_title_authors_affiliations.pdf>

21. Thank you for stating the following financial disclosure:

“Indonesia Endowment Fund/LPDP for publication support.”

Please state what role the funders took in the study.  If the funders had no role, please state: "The funders had no role in study design, data collection and analysis, decision to publish, or preparation of the manuscript."

If this statement is not correct you must amend it as needed.

Please include this amended Role of Funder statement in your cover letter; we will change the online submission form on your behalf.

Response: the funder has no role, the statement has been added in the manuscript and in the cover letter

22. Thank you for stating the following in the Acknowledgments Section of your manuscript:

“We would like to thank Poh Chua, medical librarian who helped us in literature search. The authors of this paper gratefully acknowledge Indonesia Endowment Fund/Lembaga Pengelola Dana Penelitian (LPDP) for PhD scholarship funding awarded to NDP.”

We note that you have provided funding information that is currently declared in your Funding Statement. However, funding information should not appear in the Acknowledgments section or other areas of your manuscript. We will only publish funding information present in the Funding Statement section of the online submission form.

Please remove any funding-related text from the manuscript and let us know how you would like to update your Funding Statement. Currently, your Funding Statement reads as follows:

“Indonesia Endowment Fund/LPDP for publication support.”

Please include your amended statements within your cover letter; we will change the online submission form on your behalf.

Response: Funding information has been removed from the acknowledgement section.

23. We note that your Data Availability Statement is currently as follows: [All relevant data are within the manuscript and its Supporting Information files.]

Please confirm at this time whether or not your submission contains all raw data required to replicate the results of your study. Authors must share the “minimal data set” for their submission. PLOS defines the minimal data set to consist of the data required to replicate all study findings reported in the article, as well as related metadata and methods (<https://journals.plos.org/plosone/s/data-availability#loc-minimal-data-set-definition>).

Response: minimal data set (4 Microsoft excel worksheets) have been added, namely:

- Minimal Data 1_Post COVID-19 Condition based on SARS-CoV-2 Variants (data for Figure 2).xlsx
- Minimal Data 2_Post COVID-19 Condition based on Gross National Income
- Minimal Data 3_Predominating SARS-CoV-2 Variants Between Included Studies (Data for Figure 4)
- Minimal Data 4_Risk of bias assessment of included studies (For Supplementary 2)

24. We note that Figure 3 in your submission contain [map/satellite] images which may be copyrighted. All PLOS content is published under the Creative Commons Attribution License (CC BY 4.0), which means that the manuscript, images, and Supporting Information files will be freely available online, and any third party is permitted to access, download, copy, distribute, and use these materials in any way, even commercially, with proper attribution. For these reasons, we cannot publish previously copyrighted maps or satellite images created using proprietary data, such as Google software (Google Maps, Street View, and Earth). For more information, see our copyright guidelines: <http://journals.plos.org/plosone/s/licenses-and-copyright>.

Response: The map has been redesigned to remove any copyright

The map data was obtained from the World Bank's website, specifically referring to this link: https://datacatalog.worldbank.org/search/dataset/0037712/World-Development-Indicators. These data were an open source one and were licensed under a Creative Commons Attribution 4.0, stated on the website.

We use Excel to visualize the map and later also use Canva to enhance the picture quality
